# Comparison of Clinical Outcomes Between Fluoroscopic and Computer Tomographic Guidance in Concurrent Use of Radiofrequency Ablation and Vertebral Augmentation in Spinal Metastases: A Scoping Review

**DOI:** 10.3390/diagnostics15121463

**Published:** 2025-06-09

**Authors:** Qing Zhao Ruan, Syena Sarrafpour, Jamal Hasoon, R. Jason Yong, Christopher L. Robinson, Matthew Chung

**Affiliations:** 1 Warren Alpert Medical School, Brown University, Providence, RI 02903, USA; qing_zhao_ruan@brown.edu (Q.Z.R.); syena.sarrafpour@gmail.com (S.S.); 2 Department of Anesthesia and Pain Medicine, UTHealth McGovern Medical School, Houston, TX 77030, USA; jjhasoon@gmail.com; 3 Department of Anesthesiology, Perioperative and Pain Medicine, Harvard Medical School, Brigham and Women’s Hospital, 75 Francis St, Boston, MA 02118, USA; ryong@bwh.harvard.edu; 4 Department of Pain Medicine, Division of Anesthesia, Critical Care and Pain Medicine, The University of Texas MD Anderson Cancer Center, Houston, TX 77030, USA; mchung1@mdanderson.org

**Keywords:** radiofrequency ablation, vertebral augmentation, kyphoplasty, scoping review, spinal metastases

## Abstract

**Background/Objectives**: The image guidance of choice for the combination therapy of radiofrequency ablation (RFA) and vertebral augmentation (VA) in the context of vertebral disease from spinal metastases are fluoroscopy and computer tomography (CT). Here, we aimed to assess the roles of both imaging modalities and if adoption of either would influence clinical outcomes of pain, physical function, and quality of life (QoL). RFA has been favored as a minimally invasive option for managing painful spinal metastases, and it is often coupled with VA to treat underlying osseous structural instability. This combination therapy of RFA with VA, which could be performed under CT or fluoroscopy, has in recent years been recognized as highly successful for pain control and functional restoration of metastatic spine lesions. **Methods**: Our scoping review was performed in accordance with the Preferred Reporting Items for Systematic Reviews and Meta-Analysis (PRISMA). The databases accessed were Medline and Embase, and the time frame of the search was set from database inception to 2 January 2025. The inclusion eligibility included primary research studies utilizing clearly defined imaging modalities of interest with measurable clinical end points of pain, quality of life (QoL), analgesic use, or complications. **Results**: Twenty-two articles were identified after screening fifty-eight papers using the databases. Fluoroscopy alone was the more frequently adopted imaging modality (*n* = 17/22, 77.3%). Almost all of the papers, regardless of the imaging modality used, consistently demonstrated reduction in pain, improvement in QoL, as well as a decrease in analgesia use. Complications were present but had minimal clinical implications, aside from a single article which appeared to demonstrate significantly higher cement leak rates with a singular case of resultant paraplegia. **Conclusions**: Fluoroscopy- and CT-guided RFA with VA have both proven to be efficacious in reducing patient discomfort and improving functionality while keeping risks of permanent neurological injuries to a minimum.

## 1. Introduction

Cancerous bone pain in the context of spinal metastases has a high morbidity and is associated with both functional impairment and psychological distress from excruciating pain [1]. The underlying cancerous pathophysiology can manifest with signs and symptoms reflecting inflammation, ischemia, compression, neuropathic processes, or any combination [2,3]. As cancer survival rates improve, there is an increasing need for minimally invasive and efficacious modalities of treatment aiming at both disease and symptom control. Clinical objectives are thereby focused on the preservation of neurological function, enhancement of pain control, improvement in the quality of life, and the maintenance of structural spinal stability.

Radiation therapy, including conventional external beam radiation therapy, spine stereotactic radiosurgery, and spine stereotactic body radiotherapy, are standard-of-care treatments for vertebral body metastases without metastatic spinal cord compression (MSCC) [4,5]. However, adverse events such as radiation myelopathy and vertebral compression are not uncommon complications [5,6]. Surgical intervention aims to alleviate pain through nerve decompression and structural stabilization. The downside of surgery is its invasiveness and is typically reserved for radioresistant tumors with significant MSCC [7,8].

Radiofrequency ablation (RFA) has been utilized as a minimally invasive option for managing painful vertebral body metastases [9,10]. However, due to the fragility of diseased bone, RFA alone is unable to restore structural integrity of the spine. Therefore, it is often coupled with vertebral augmentation (VA) procedures such as percutaneous vertebroplasty (PVP) or kyphoplasty (PKP) to address the underlying structural compromise [4,11,12,13,14]. Both VA procedures involve polymethylmethacrylate (PMMA) bone cement injection into an osseous fracture site to achieve mechanical stabilization. PKP additionally involves insertion of a balloon catheter into the fractured vertebra, creating a cavity for subsequent PMMA instillation [15]. This combination therapy of RFA with VA, both image-guided, has in recent years been recognized as a highly successful treatment modality for pain control and functional restoration in metastatic spine lesions [4,10,11,16,17,18,19,20,21].

The image guidance of choice for the combination therapy of RFA and VA are fluoroscopy and computer tomography (CT). Given the disparate nature of these imaging techniques in clinical application and resource consumption, it is prudent to clarify and consolidate the available evidence in the existing literature in the performance of this combination therapy. Our scoping review seeks to establish the roles of both imaging modalities and how adoption of either would influence clinical outcomes of pain, physical function, quality of life (QoL), analgesia use, and procedure-associated complications.

## 2. Methods

Our scoping review was performed in accordance with the criteria defined by Preferred Reporting Items for Systematic Reviews and Meta-Analysis (PRISMA). Medline and Embase were queried for the literature search, and the time frame of the search was set from database inception to 2 January 2025. The determination of the search strategy was carried out by two authors (QR and CR), and it was subsequently reviewed and approved by all authors following necessary amendments. Screening of the articles and inclusion for review were collectively performed by authors QR and CR, and in the event of disagreement, a third author was consulted for opinion. Data charting and summary were then completed in unison on calibrated templates determined prior to article selection. The data points collected included article publication year, country where the study was performed, type of study, patient number, age of the study population, gender ratio, primary tumor location, study treatment comparison, cement volume injected in VA, supplemental conventional cancer treatment, pain intensity improvement, analgesia quantity used, functional improvement, tumor progression, follow-up duration, complications, and study conclusions. Critical appraisal was not performed on each article in concordance with the checklist under PRISMA Extension for Scoping Reviews (PRISMA-ScR). There was no funding involved.

### 2.1. Search Strategy and Selection Criteria

A comprehensive literature search was performed on 2 January 2025 on PubMed, using the following search strategy: (“radiofrequency ablation” OR “coblation”) AND (“vertebral augmentation” OR “kyphoplasty” OR “vertebroplasty”) AND (“spinal cancer” OR “spinal metastases”). A similar strategy was used to query Embase.

### 2.2. Inclusion Criteria

The following inclusion criteria were selected: (1) studies with titles, articles, and abstracts written in the English language; (2) peer-reviewed primary clinical studies on therapeutic combined RFA and VA in the treatment of spinal neoplasms and metastases; (3) studies including patients ≥18 years of age; and (4) studies clearly defining the modality of imaging used as either fluoroscopy or computer tomography.

### 2.3. Exclusion Criteria

The following exclusion criteria were used: (1) articles not written in English, (2) non-human animal studies, (3) individuals ≤ 18 years of age, (4) abstracts without full-text articles submitted for conferences or in supplementary sections of journals, (5) non-original review articles, (6) studies without clearly defined imaging modality used in their interventions, (7) articles without pain or functional outcomes, (8) concurrent surgical treatments performed, and (9) studies adopting a combination of fluoroscopy and computer tomography or CT–fluoroscopy for the procedure.

## 3. Results

**Figure 1:**
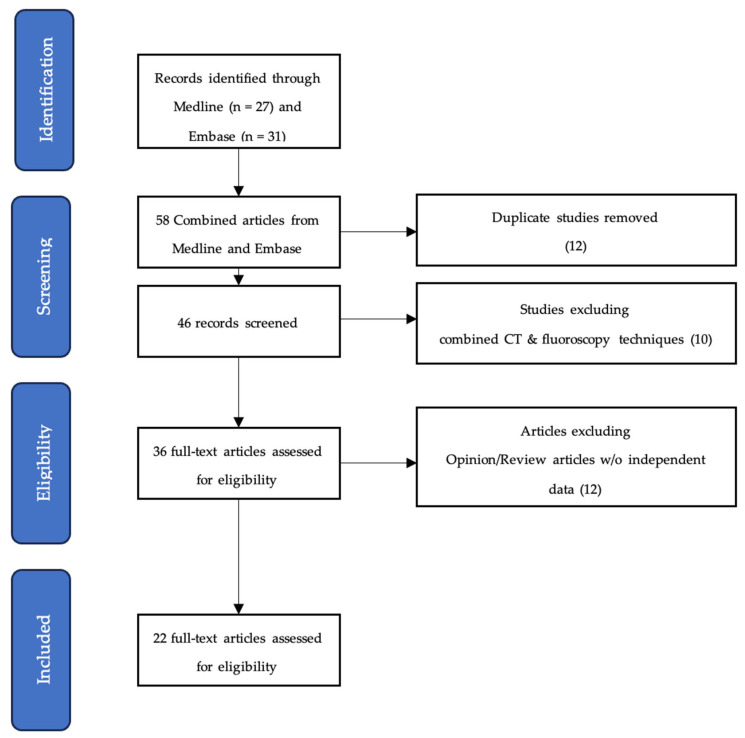
PRISMA flowchart overview of scoping review analysis.

A total of 22 unique articles were identified using the databases previously defined (Figure 1 and Table 1 and Table 2) [16,19,21,22,23,24,25,26,27,28,29,30,31,32,33,34,35,36,37,38,39,40]. The selected articles were published between 2004 and 2023, and their origins were distributed between North America, Europe, and Asia. Fluoroscopy alone was the more frequently adopted imaging modality for the combination RFA and VA treatment (*n* = 17/22, 77.3%). Retrospective studies (*n* = 17/22, 77.3%) made up most of the screened literature, and the follow-up duration ranged from 2 weeks to 36 months. All articles reported at least one of the measurable clinical end points indicating degree of pain control (most commonly visual analogue scale (VAS) and numerical rating scale (NRS)), level of physical function (most commonly Oswestry Disability Index (ODI)), or quantity of analgesics used.

### 3.1. Pain Control

#### 3.1.1. CT Guidance Only

Five papers demonstrated significant pain relief following treatment with combined RFA and VA [19,37,38,39,40]. All pain scores reported ranged from 0 to 10. Proschek et al. reported a mean VAS reduction from 7.6 to 5.0 post-treatment (*p* = 0.005), and further reductions to 3.5 (*p* = 0.005) were reported at 15 to 36 months after combined RFA and VA treatment [37]. Madaelil et al. reported reductions in numerical pain scores ranging from 9 to 3 in seven patients who went through combined RFA and VA over a follow-up duration ranging from 0.9 to 14.4 months [38]. Similarly, the combined VAS score in Zhao et al.’s cohort, where the majority of the patients (*n* = 13/16) underwent combined RFA and VA, revealed immediate VAS reductions from 8.1 ± 1.4 (range 5.0–10) to 5.5 ± 1.1 (range 3.4–7.8) (*p* < 0.05) at 24 h following the procedure [39]. This trend continued up to 6 months, with VAS decreasing to 1.4 ± 0.8 (range 0–2.5) (*p* < 0.01). This steady reduction in VAS was also mirrored by Kastler et al.’s study, where pre-operative mean VAS at 8.4 ± 1.4 decreased to 2.2 ± 2.4 immediately at 24 h and was maintained at 1.8 ± 2.1 at 12 months after treatment [40]. This was also in line with the results of Pusceddu et al., who reported VAS of 8.0 (IQR 7.0–8.0) pre-procedure and 0.5 (IQR 0.0–2.0) just 1 week post-treatment. This was seen to decrease further at follow-ups at 1, 3, and 6 months [19].

#### 3.1.2. Fluoroscopy Guidance Only

There were significant reductions of pain in all fluoroscopy guidance only studies that reported pain scores (*n* = 16/17) [16,21,22,23,24,25,26,27,28,29,30,31,32,33,34,36]. Abdelgawaad et al. demonstrated a mean reduction in VAS from 7.2 ± 2.3 (range 4–9) down to 2.7 ± 1.9 (range 1–5) on day 3 after treatment [22]. This reduction in pain was maintained at 6 months, with a mean VAS of 3 ± 2.1 (range 1–6) (*p* < 0.0001). Similarly, Gu et al. reported significant and sustained reductions in VAS from 7.09 ± 0.94 pre-procedure to 2.30 ± 1.03 (*p* < 0.05) at 1 month, and 2.03 ± 1.14 (*p* < 0.05) and 1.90 ± 1.23 (*p* < 0.05) 6 months [24]. In fact, this reduction in VAS was maintained at 1 year at 1.72 ± 1.35 (*p* < 0.05) and over 1 year at 1.88 ± 1.54 (*p* < 0.05). Zheng et al. revealed a similar pattern, with improvement in VAS from 7.69 ± 1.12 (*p* < 0.01) to 2.96 ± 0.92 (*p* < 0.05) at 6 months [34]. Jain et al. demonstrated a similar result, with improvements in VAS in both groups utilizing combined RFA and VA therapies [25]. The respective average pre-operative pain scores were 6.9 for SpineSTAR (South Jordan, UT, USA, Merit Medical Systems), 6.3 for isolated kyphoplasty, and 6 for OsteoCool (Minneapolis, MN, USA, Medtronic). Post-operative average pain scores decreased to 2.7 for SpineSTAR, 2.3 for isolated kyphoplasty, and 1.7 for OsteoCool. Although the patients were followed for a short time period, Sandri et al. revealed mean VAS improvements from 8.0 to 1.8 at 72 h and 1.9 at 6 weeks post-operatively (*p* < 0.001) [31]. Yildizhan et al. displayed a reduction in VAS in the RFA and vertebroplasty group from 7.44 ± 1.06 to 2.31 ± 1.42 (*p* < 0.001) [33].

### 3.2. Quality of Life (QoL)/Functional Capacity

#### 3.2.1. CT Guidance Only

Three articles measured QoL changes following treatment. Proschek et al. reported a reduction in mean ODI from 66% to 36% (*p* = 0.003) immediately after combined RFA and VA, and this was sustained for 15 to 36 months, with mean ODI reported to be 35% (range 26–38%, *p* = 0.071) [37]. Zhao et al. used the European Organization for Research and Treatment of Cancer QoL Questionnaire (9EORTC QLQ C-30) scale and revealed improved physical function (*p* = 0.03), emotional function (*p* = 0.003), insomnia (*p* = 0.002), and global health status (*p* = 0.002) 1 month after treatment [39]. The functional mobility scale (FMS) improved significantly (*p* = 0.002) after 1 month of treatment in Pusceddu et al.’s study, where patients that reported wheelchair use (*n* = 4/8) before treatment improved to limited painful ambulation and even normal ambulation (*n* = 4/8) [19].

#### 3.2.2. Fluoroscopy Guidance Only

The studies which analyzed QoL and ODI all demonstrated positive outcomes (*n* = 9/17) [16,21,24,26,28,29,30,33,36]. Gu et al. revealed significant improvements in ODI from 58.84 ± 2.34 to 22.46 ± 4.91 (*p* < 0.05) at 1 month, 19.66 ± 5.93 (*p* < 0.05) at 3 months, 20.41 ± 9.41 (*p* < 0.05) at 6 months, and 22.05 ± 11.94 (*p* < 0.05) at over a year [24]. ODI improved in Yildizhan et al.’s study, which demonstrated an ODI reduction from 78.5% to 14.2% post-treatment (*p* < 0.001) [33]. General activity levels increased in 81% of patients in Greenwood et al.’s analysis, and Munk et al. reported improvements in mobility in 18/19 patients studied [16,28]. Nilgun et al. revealed that quality of life improved, with the mean pre-procedural ODI being 71.04, which was reduced to mean scores of 27.46, 28.50, 29.00, and eventually 34.00 at 1, 2, 3, and 6 months, respectively, with *p* < 0.05 at all time points with respect to the original pre-procedural score [29]. Similarly, Reyes et al. demonstrated ODI improvement from 34.9 ± 18.3 to 21.6 ± 13.8 after treatment (*p* < 0.0001) [30]. Lv et al. reported a comparable trend, with ODI reduction from 77.52 ± 8.84 to 46.46 ± 6.46 for patients who underwent RFA and bone cement (*p* < 0.05) [26].

### 3.3. Analgesia Use

#### 3.3.1. CT Guidance Only

Only two studies in this group reported on analgesic use before and after combined RFA and VA therapies. Zhao et al. summarized that the analgesic doses consumed were reduced in all their patients following the procedure and were completely stopped at 2 months [39]. Madaeilil et al. reported mixed results of increased analgesia use in 4 out of their 10 patients following the combined RFA and VA [38].

#### 3.3.2. Fluoroscopy Guidance Only

Four out of the seventeen fluoroscopy guidance only studies assessed patient analgesia use [16,25,28,31]. Jain et al. followed 64 patients who underwent combined RFA and VA via two different RFA systems (OsteoCool and SpineSTAR) or VA alone, recording opioid intake 1 month after therapy [25]. The frequency of opioid usage was deemed similar between all three groups (*p* = 0.82). Greenwood et al. revealed a reduction in opioid use in 62% (13 of 21) of subjects at 4 weeks, while 19% (4 of 21) had either an increase or no change in opioid consumption at 4 weeks after the combination therapy of RFA and VA [16]. Munk et al. similarly showed a reduction in opioid analgesic use in 18 out of 19 patients at 12–20 weeks after treatment [28]. In 17 patients, there was a decreased frequency of opioid use, while in one patient, there was complete cessation of opioid use. In all 11 patients assessed by Sandri et al., it was reported in descriptive text that all of them had reductions in analgesia use following treatment [31].

### 3.4. Complications

#### 3.4.1. CT Guidance Only

Two articles in the CT guidance group explicitly reported on complication rates [39,40]. Zhao et al. described a single case of cement leakage through a tumor-eroded vertebral body posterior cortex, where the leaked content transiently compressed nerve roots [39]. The leaked cement was subsequently surgically removed with complete resolution of symptoms in 2 days. Kastler et al. reported the identification of asymptomatic cement leakage on 11 of 16 post-operative CT scans [40].

#### 3.4.2. Fluoroscopy Guidance Only

Nine of the seventeen articles reported on procedure-related complications [16,22,24,26,28,29,31,35,36]. Wang L et al. reported their pulmonary cement embolization rate to be 23.4% and that the significantly higher risks of embolization were attributed to thoracic lesions (*p* = 0.0008) and cement leakage identified intraoperatively (*p* = 0.017) [35]. However, none of the 11 patients suffered from any clinical manifestation of these complications.

Abdelgawaad et al. described five incidents of cement leakage into needle tracks, draining veins, and the disc space without neurological complications in the context of 75 independent procedures [22]. Munk et al. reported minor cement leaks in six patients and an episode of transient thermal sciatic nerve injury, which was self-limiting, lasting 48 h [28]. Nilgun et al. similarly described two transient episodes of motor deficits without evidence of cement leakage and one case of pulmonary embolism with what was described as “mild” symptoms in text [29]. Gu et al. demonstrated that in the combined RFA and VA group, the cement leakage rate was 41.4% (29 of 70) [24]. The breakdown of complications revealed 7 leaks into disc spaces, 3 into needle tracks, 5 into paravertebral spaces, and 14 into veins. Lv et al. revealed that in the combined RFA and VA group in their study, the cement leakage rate was 6.4% [26]. Wang F et al. reported a vascular cement leak frequency of 6 out of 17 procedures and a non-vascular cement leak frequency also at 6 out of 17 cases in their study [36].

## 4. Discussion

Our scoping review explored multiple facets of image-guided spinal intervention in the form of combined RFA and VA in the context of metastatic spinal disease, analyzing fluoroscopy- and CT-guided procedures. From the number of articles extracted, fluoroscopy appeared to be more frequently adopted as the modality of choice (17 of 22), likely given the relative ease of access and reduced technical and medical resources utilized. However, both imaging modalities consistently demonstrated favorable outcomes relative to control via measures of pain scores, QoL indices, and supplemental analgesic consumption.

Of the papers evaluated, Sandri et al. demonstrated the lowest post-procedure pain score, where main VAS reduced from 8.0 to 1.8 at 72 h and 1.9 at 6 weeks in the fluoroscopy-guided imaging group [31]. Similarly for CT-guided therapy, Zhao et al. showed post-procedural VAS reduction from 8.1 to 1.4 over 6 months, which is also supported by the results of Pusceddu et al. [19,39]. The ODI in Yildizhan et al.’s article reduced to 14.2% from 78.5% after fluoroscopy-guided treatment, while in the CT group, Proschek et al. showed ODI improvement from 66% to 35% at 6 months [33]. These findings were in alignment with that in the existing literature in establishing the effectiveness of combined RFA and VA in improving clinical outcomes [41].

Complications from combined RFA and VA employing both modalities were clinically insignificant, although it was recognized that transient radiculitis and cement leakage into tissues, needle tracks, and even pulmonary vasculature were not uncommon occurrences. Paralysis was only reported in one article [24]. The description of operative steps appeared to differ to some extent from that of articles published in the West, where the author performed sequential dilation of the initial needle tract with a specialized working cannula (Dragon-Crown Co, Jinan, China), followed by trephine insertion to chip away at the osseous pedicle prior to cement instillation [24]. Whether the more invasive nature of this technique led to the higher rate of complications would require further studies in the future.

### 4.1. Precision

There is currently no literature comparing the therapeutic accuracy of ablative tumor therapy in the spine with subsequent spinal augmentation performed under different imaging modalities. Feigl et al. compared the accuracy of needle placement for routine medial branch radiofrequency ablation under fluoroscopy and CT guidance in a cadaveric study where the exact needle positions were ascertained via meticulous dissection in a cadaver laboratory [42]. The results revealed that needle placement under fluoroscopy more precisely aligned the electrode trajectory with the long axis of the target nerves. This result however was deemed unique to this procedure as fluoroscopy allowed for a cranio-ventral direction of electrodes in line with the medial branches, while replicating this trajectory would prove challenging under CT guidance. Therefore, these results could not be generalized to all ablation procedures [42].

Similarly, others have performed comparisons of precision in transpedicular biopsy of spinal lesions using fluoroscopy versus CT guidance, which could be used as a proxy to understanding the accuracy of transpedicular vertebral augmentation techniques [43]. For biopsies, the needle was delivered through the pedicle toward the target site with gentle tapping with a mallet under imaging guidance. Once sufficiently close in proximity to the target site, the trocar was pulled back and cannula advanced over the target location, where aspiration of the specimen was performed through a syringe, and the tissue sample was collected using a stylet. There was no significant difference between diagnostic accuracy attained using fluoroscopy compared to CT (*p* = 0.670), and the procedural duration was no different (16.0 ± 7.5 min, *p* = 0.946). The results could therefore be extrapolated to acknowledge that fluoroscopy- and CT-guided transpedicular interventions in the vertebral body could be very similar in accuracy.

In single-stage kyphoplasty, the combined use of CT with fluoroscopic guidance appeared to allow proceduralists to obtain better visualization of the target pedicle and tighter monitoring of osteointroducer insertion and balloon positions within the vertebral body compared to a fluoroscopy-only technique [44]. Amorettie et al. demonstrated the above via a single-institution study of 41 kyphoplasty procedures where two experienced interventional radiologists graded the image quality and technique precision obtained during kyphoplasty utilizing the two different techniques [44]. Despite superior pedicle visualization and balloon position under combined CT and fluoroscopy guidance, cement distribution and leakage complication were no different between the two, indirectly suggesting that the clinical outcomes between the two groups were minimal. Extrapolating this result to our review, where the initial delivery of the radiofrequency ablation probe was similar to osteointroducer insertion in kyphoplasty, one could argue a higher degree of imaging precision achieved with CT guidance. Whether this directly translated to improved therapeutic outcome is less clear.

### 4.2. Radiation Dose

Conventional understanding of CT-guided procedures suggested significantly higher dose of radiation exposure utilizing the latter. Lee et al. demonstrated that performing a transpedicular biopsy procedure which takes less than 20 min per case regardless of technique, radiation exposure to patients was 26 times higher and provider exposure was 2 times more significant in the CT group compared to the fluoroscopy group [43].

However, ultra-low-dose CT techniques had been devised for procedures proven to undercut even fluoroscopy radiation doses for routine interventional pain procedures. Wieschhoff et al. described this technique in their study on lumbar epidural steroid injections [45]. They reported significantly lower radiation exposure in the CT–fluoroscopy group compared to the fluoroscopy group (0.15 mSV ± 0.11 vs. 0.30 mSv ± 0.34) (*p* < 0.001). For this reduction in radiation exposure, there was no compromise on procedure time (both procedures averaged approximately 21 min) or the post-procedure clinical outcome (mean pain score reduction 3.3 ± 2.9 vs. 3.9 ± 2.6, *p* = 0.16) [45].

#### Novel Imaging Modalities

In addition to CT–fluoroscopy being a relatively novel approach to performing spinal interventions, as described by Wieschhoff et al., techniques adopting augmented reality have also been reported [45]. Sag et al. described fluoroscopy-guided sacroplasty with augmented reality overlay, comparing it with CT–fluoroscopy guidance in their study of 12 patients from 2019 to 2020 [46]. This was achieved through an FDA-approved software, Cone Beam CT Augmented Fluoroscopy (CBCT-AF), which made it possible to provide real-time enhanced fluoroscopy with virtual overlay, including physician-defined contours over margins of clinical interest, establishing unique vectors for accurate instrumental navigation, thereby creating an augmented-fluoroscopy experience. The authors noted that there was no significant difference in radiation dose (*p* = 0.20), total anesthesia time (*p* = 0.71) or total volume of cement infused (*p* = 0.21). Post-operative improvement in pain appeared similar, with VAS reducing by 6.14 and 5.25 in the CT and augmented reality groups, respectively (*p* = 0.46) [46].

## 5. Limitations

Our study is limited by its nature of being a scoping review. Statistical analyses were not performed to critically assess outcome disparity for procedures performed under the two different imaging modalities. However, sufficient variabilities existed in the methods and clinical contexts of the studies that would make head-to-head comparisons challenging in a patient population with widely disparate follow-up durations. The definition of technical precision is difficult to assess, and the ability to instill larger volumes of cement for VA does not directly translate to clinical success given the known and widely reported risks of leak.

## 6. Conclusions

Fluoroscopy- and CT-guided combined RFA and VA have both proven to be efficacious in reducing pain and improving functionality, at the same time displaying a track record of clinical safety. Statistical superiority of one over the other in our measured end points falls outside the scope of this article, and future studies could further explore these elements in depth, taking into consideration the advent of newer and safer modalities of precision imaging.

## Figures and Tables

**Table 1:** Characteristics and clinical outcomes of the studies using only CT guidance for combined RFA and VA. EORTC QLQ: European organization for treatment of cancer quality of life questionnaire; FMS: functional mobility scale; ODI: Oswestry index; QoL: quality of life; RFA: radiofrequency ablation; VA: vertebral augmentation.

Articles (Year)	Study Country	Study Type	Patient No.	Age (Range)	Male–Female Number	Tumor Location	Treatment Group(s)	Mean Cement Volume	Supplemental Treatment	Pain Improvement	Analgesia Use	Functional/QoL Improvement	Recurrence/Progression	Follow-Up Duration	Complications	Conclusion
Proschek (2009) [37]	Germany	Prospective	16	Mean: 59.5 years (52–69)	0:16	Thoracic and lumbar spine	RFA alone vs. RFA with VA	Unreported	Unreported	Pain reduction (~50%) in both groups	Unreported	Improved QoL (ODI)	No local recurrence	Mean: 20.4 months	None reported	Safe, no additional benefit of cement in pain/QoL
Madaelil (2016) [38]	USA	Retrospective	11	Median: 58 years (37–79)	3:8	Sacrum	RFA alone vs. RFA with VA	9.1 mL	45% received pre-procedural or post-procedural chemotherapy	NRS reduced from 8 to 3 at 1 month	Increased in 40%	Not measured	75% local control	Median: 4.7 months (0.9–28.7)	None reported	RFA safe and effective for sacral metastases
Zhao (2018) [39]	China	Retrospective	16	Mean: 66.8 years (54–84)	4:12	Sternum, scapula, ribs, thoracic spine, lumbar spine, and sacrum	RFA alone vs. RFA with VA	Unreported	Unreported	VAS reduced from 8.1 to 1.4 over 6 months	Stopped within 2 months	Improved QoL (via EORTC QLQ-C30)	No recurrence (6–12 months)	6–12 months	1 case (6.25%) of cement leakage	Safe and effective for palliative treatment
Kastler (2021) [40]	France	Prospective	25	Mean: 60 years	18:7	Thoracic spine, lumbar spine, sacrum, and coccyx	RFA alone vs. RFA with VA	4 mL	Unreported	VAS improvement up to 79%	Unreported	Not measured	Unreported	Up to 12 months	Minor cement leakage (11/16) without clinical symptoms	Effective, well-tolerated under local anesthesia
Pusceddu (2023) [19]	Italy	Retrospective	16	Mean: 67 years (41–84)	8:8	Thoracolumbar spine	RFA, cavity creation, vertebral augmentation	Unreported	Unreported	Significant reduction in VAS (*p* < 0.001)	Unreported	Improved mobility (FMS)	No tumor recurrence	6 months	None reported	Safe and effective for pain and QoL improvement

**Table 2:** Characteristics and clinical outcomes of the studies using only fluoroscopy guidance for combined RFA and VA. FACT: functional assessment of cancer therapy; FMS: functional mobility scale; ODI: Oswestry index; QoL: quality of life; RFA: radiofrequency ablation; VA: vertebral augmentation.

Articles (Year)	Study Country	Study Type	Patient No.	Age (Range)	Male–Female Number	Tumor Location	Treatment Group(s)	Mean Cement Volume	Supplementary Treatment	Pain Improvement	Analgesia Use	Functional/QoL Improvement	Recurrence/Progression	Follow-Up Duration	Complications	Conclusion
Abdelgawaad (2021) [22]	Germany	Retrospective	60	(35–80)	35:25	Thoracic and lumbar spine	Cooled RFA, VA	Unreported	Radiotherapy and chemotherapy	Mean VAS reduced from 7.2/10 ± 2.3 (4–9) to 2.7/10 ± 1.9 (1–5) on day 3 and 3/10 ± 2.1 (1–6) at 6 months, *p* = 0.0001	Unreported	Unreported	No recurrence	Mean 13.2 ± 6.3 months; minimum 6 months; maximum 36 months	2 leaks into needle tracks, 2 leaks into veins, and 1 into disc spaces—all asymptomatic	Combined RFA and VA appears to be a safe, practical, effective and reproducible palliative treatment
Georgy (2009) [23]	USA	Retrospective	37	(45–75)	16:21	Thoracic and lumbar spine	RFA with VA	Unreported	Unreported	VAS improved in 89.5% of patients, mean VAS reduced from 7.9 to 4.2 (*p* < 0.0001)	Unreported	Unreported	Unreported	Median 6 months	Unreported	Effective alternative for advanced metastases
Greenwood (2015) [16]	USA	Retrospective	21	Mean: 61.8 (30–84)	13:9	Undefined spinal location	RFA/cryoablation, VA, radiation therapy	Unreported	Radiation therapy	Pain scores reduced from (8.0, SD = 2.3) pre-operatively to 2.9, SD = 3.3; *p* < 0.0003	Opioid use was decreased in 62% (13/21), remained unchanged in 19% (4/21), and increased in 19% (4/21) at 4 weeks	General activity level at 4 weeks after ablation treatments was increased in 81% (17/21) and decreased in 19% (4/21)	Disease stable in 12/13 patients at 3 months and 10/10 patients at 6 months	6 months	No complication other than undefined post-procedural infection	Safe and effective for radiation-resistant tumors for control of pain and local control of tumor
Gu (2017) [24]	China	Prospective	124	Mean: 58.2 (37–76) vs. 59.5 (33–90)	75:49	Thoracic and lumbar spine	Interventional tumor removal, RFA, and VA	Unreported	Unreported	Significant reduction in VAS at 1, 3, and 6 months, and >1 year (*p* = 0.05)	Not reported	Significant improvement in ODI at 1, 3, and 6 months, and >1 year (*p* = 0.03)	Minimal	6–12 months	29 and 29 cement leaks in 70 and 53 vertebral bodies in groups A and B, respectively; 1 severe complication of paraplegia	Effective for spinal stability and pain relief
Jain (2020) [25]	USA	Retrospective	64	Mean: 62.3	Undefined	Thoracic and lumbar spine	RFA (Osteocool) and VA vs. RFA (SpineSTAR) and VA vs. VA only	Unreported	Radiation and chemotherapy	VAS improvement in all groups; re-operative pain scores were on average 6.9/10 for SpineSTAR, 6.3/10 for kyphoplasty alone, and 6/10 for OsteoCool; post-operative pain scores were on average 2.7/10 for SpineSTAR, 2.3/10 for kyphoplasty alone, and 1.7/10 for OsteoCool	Narcotic usage within post-operative month one was seen in 11/22 (50%) of SpineSTAR cases, 9/30 (30%) of kyphoplasty cases, and 5/12 (41.7%) of OsteoCool cases	Unreported	Unreported	1 month	Unreported	Comparable outcomes for both methods; overall decrease in pain scores for all treatment groups; however, there was no substantial difference in pain scores between patients who received RFA with vertebral augmentation vs. those who received kyphoplasty alone
Lv (2020) [26]	China	Retrospective	87	Mean: 64	53:34	Thoracic and lumbar spine	VA alone vs. RFA with VA	Unreported	Unreported	VAS for groups A and B improved from 7.52 ± 1.44 and 7.63 ± 1.52 to 2.23 ± 0.46 and 3.15 ± 0.52, respectively, at 6 months	Unreported	Improved QoL; ODI for groups A and B improved from 77.52 ± 8.84 and 76.65 ± 8.12 to 46.46 ± 6.46 and 52.15 ± 7.52, respectively, at 6 months (*p* = 0.04)	11.4% recurrence in group A and 30.8% in group B	6 months	6.4% cement leak in group A and 20.5% cement leak in group B, all asymptomatic	Bone cement combined with RFA in the treatment of spinal metastatic tumor can effectively relieve patients’ pain, improve their ability for daily activities, and enhance the spinal stability
Masala (2004) [27]	Italy	Prospective	3	Mean: 72.3 (63–82)	1:2	Spine (undefined)	RFA with VA	Unreported	Unreported	VAS improved significantly; reduction from average of 8.6 points of VAS pre-procedure to 2.6 post-procedure	Not reported	Unreported	Unreported	6 months	Unreported	Safe and effective for VCF
Munk (2009) [28]	Canada	Retrospective	19	Mean: 58.9 (42–82)	5:14	Spine, pelvis, and long bones	RFA with VA	6.1 mL	Unreported	VAS reduced from 7.90 (range, 7.0 –9.0) to 3.82 (range, 0.0–6.2) (*p* < 0.0001)	Analgesia use reduction achieved in 18 patients, with complete cessation in 1 patient	18 patients had improvement in mobility	Unreported	Median 9 months	7 minor complications; 6 cement extravasations, 1 thermal nerve injury	Effective for pain relief in neoplastic lesions
Nilgun (2022) [29]	Turkey	Retrospective	41	Mean: 67 (45–87)	22:19	Thoracic and lumbar spine	RFA with VA	Unreported	Unreported	VAS reduced significantly; mean VAS score was 7.4 at the preprocedural assessment and 3.2 at 6 months (*p* < 0.0001)	Unreported	Improved QoL; mean ODI was 71.04 at the preprocedural assessment and 34 at 6 months	Unreported	6 months	1 patient had pulmonary embolism (mild symptoms), 2 patients had transient motor deficits without cement leak	Safe and effective for metastatic spinal pain
Reyes (2017) [30]	USA/Europe	Multicenter	49	64.3 ± 12.6	15:34	Thoracic and lumbar spine	RFA with VA	Unreported	5 patients had radiotherapy and 3 had chemotherapy	VAS improved from 7.9 ± 2.5 (range 2–10) to 3.5 ± 2.6 (range 0–10) (*p* < 0.0001)	Unreported	ODI improvement; pre-procedure ODI 34.9 ± 18.3 (range 13–50) vs. post-procedure ODI 21.6 ± 13.8 (range 0–45) (*p* < 0.0001)	1 case of recurrence	2–4 weeks	None reported	Effective for pain reduction and functional improvement
Sandri (2010) [31]	Italy	Retrospective	11	Mean: 68 (58–82)	2:9	Cervical, thoracic, and lumbar spine	RFA with VA	Unreported	Unreported	Mean VAS reduced from 8.0 to 1.8 at 72 h, and 1.9 at 6 weeks (*p* < 0.001)	Reduction in use in all patients	Unreported	No complications	6 weeks	1 case of asymptomatic cement leakage	Safe and effective for osteolytic metastases
Sayed (2019) [21]	USA	Prospective	30	18+	19:11	Thoracic and lumbar spine	RFA with VA	Unreported	Unreported	NRS-11 improved from 5.77 to 2.61 at 3 months, *p* < 0.01	Unreported	Improved QoL; FACT-G7 improved from 13.0 at baseline to 15.11 at 3 months, *p* = 0.07	Unreported	3 months	Non reported complication	Safe and effective for metastatic spinal lesions
Tomasian (2018) [32]	USA	Retrospective	27	(23–86)	17:10	Thoracic and lumbar spine, and sacrum	RFA with VA	Unreported	Unreported	VAS not reported, but local tumor control achieved in 96% of cases	Unreported	Unreported	Local tumor control for 96% patients	16 weeks	None reported	Safe and effective for tumor control
Wang F (2021) [36]	China	Retrospective	35	Mean: 63.1 (45–83) vs. 61.5 (41–81)	22:13	Thoracic and lumbar spine	VA alone vs. RFA with VA	5.95 mL	26 patients on chemotherapy	VAS improved significantly in both groups; VAS scores of group A (1.86 ± 0.78) were significantly lower than those in group B (4.59 ± 1.06) 6 months after the treatment (*p* < 0.001)	Unreported	Significant ODI improvement in RFA + vertebroplasty group compared to single vertebroplasty (*p* < 0.05)	Unreported	6 months	No major complications; minor pain, cement leakage, pulmonary venous cement leak without symptoms	Better outcomes with combined treatment
Wang L (2023) [35]	China	Retrospective	47	Mean: 59.9 (61.5–58.3)	19:28	Thoracic and lumbar spine	RFA with VA	Unreported	Unreported	VAS score measured pre-operatively only	Unreported	Unreported	Unreported	Unreported	Pulmonary cement embolism was detected in 11 patients (23.4%), and all patients were asymptomatic	Safe with awareness of embolism risk
Yildizhan (2021) [33]	Turkey	Retrospective	66	Undefined	Undefined	Thoracic and lumbar spine	RFA alone vs. RFA with VA	Unreported	Unreported	Significant pain improvement in both groups; VAS in RFA + VP group improved from 7.44 ± 1.06 to 2.31 ± 1.42, while VAS in RFA group improved from 8.33 ± 1.07 to 4.42 ± 1.08 (*p* < 0.001)	Unreported	ODI improved from 78.5% to 14.2% post-treatment (*p* < 0.001)	No recurrence noted	6 months	None reported	Combined therapy more effective
Zheng (2014) [34]	China	Retrospective	26	Mean: 59.3 (32–75)	12:14	Thoracic and lumbar spine	RFA with VA	6.73 mL	Unreported	VAS reduced significantly; VAS improved from 7.69 ± 1.12 at baseline to 2.96 ± 0.92 at 6 months (*p* < 0.01)	Unreported	Unreported	No tumor recurrence	8.4 months	None reported	Safe and effective for palliation

## Data Availability

Not applicable.

## References

[1] Portenoy R.K., Payne D., Jacobsen P. (1999). Breakthrough pain: Characteristics and impact in patients with cancer pain. Pain.

[2] Jing D., Zhao Q., Zhao Y., Lu X., Feng Y., Zhao B., Zhao X. (2023). Management of pain in patients with bone metastases. Front. Oncol..

[3] Bollen L., Dijkstra S.P.D., Bartels R.H.M.A., de Graeff A., Poelma D.L., Brouwer T., Algra P.R., Kuijlen J.M.A., Minnema M.C., Nijboer C. (2018). Clinical management of spinal metastases—The Dutch national guideline. Eur. J. Cancer.

[4] Colonna S., Bianconi A., Cofano F., Prior A., Di Perna G., Palmieri G., Zona G., Garbossa D., Fiaschi P. (2023). Radiofrequency Ablation in Vertebral Body Metastasis with and without Percutaneous Cement Augmentation: A Systematic Review Addressing the Need for SPINE Stability Evaluation. Diagnostics.

[5] Sahgal A., Myrehaug S.D., Siva S., Masucci G.L., Maralani P.J., Brundage M., Bulter J., Chow E., Fehlings M.G., Foote M. (2021). Stereotactic body radiotherapy versus conventional external beam radiotherapy in patients with painful spinal metastases: An open-label, multicentre, randomised, controlled, phase 2/3 trial. Lancet Oncol..

[6] Guckenberger M., Dahele M., Ong W.L., Sahgal A. (2023). Stereotactic Body Radiation Therapy for Spinal Metastases: Benefits and Limitations. Semin. Radiat. Oncol..

[7] Mossa-Basha M., Gerszten P.C., Myrehaug S., Mayr N.A., Yuh W.T., Jabehdar Maralani P., Sahgal A., Lo S.S. (2019). Spinal metastasis: Diagnosis, management and follow-up. Br. J. Radiol..

[8] Laufer I., Rubin D.G., Lis E., Cox B.W., Stubblefield M.D., Yamada Y., Bilsky M.H. (2013). The NOMS Framework: Approach to the Treatment of Spinal Metastatic Tumors. Oncologist.

[9] Wray J.K., Dixon B., Przkora R. (2024). Radiofrequency Ablation.

[10] Wallace A.N., Tomasian A., Vaswani D., Vyhmeister R., Chang R.O., Jennings J.W. (2016). Radiographic Local Control of Spinal Metastases with Percutaneous Radiofrequency Ablation and Vertebral Augmentation. Am. J. Neuroradiol..

[11] Giammalva G.R., Costanzo R., Paolini F., Benigno U.E., Porzio M., Brunasso L., Basile L., Guli C., Pino M.A., Gerardi R.M. (2022). Management of Spinal Bone Metastases with Radiofrequency Ablation, Vertebral Reinforcement and Transpedicular Fixation: A Retrospective Single-Center Case Series. Front. Oncol..

[12] Jha R.M., Hirsch A.E., Yoo A.J., Ozonoff A., Growney M., Hirsch J.A. (2010). Palliation of compression fractures in cancer patients by vertebral augmentation: A retrospective analysis. J. Neurointerv. Surg..

[13] Hirsch J.A., Hirsch A.E., Jha R., Growney M., Rabinov J.D., Nogueira R.G., Pryor J.C., Yoo A.J. (2010). Practical management of malignant compression fractures. J. Neurointerv. Surg..

[14] Lu M., Lei Z., Dai S., Hou C., Du S., Chen W., Li H. (2021). A narrative review of the application of radiofrequency ablation in the surgery of spinal metastases. Ann. Jt..

[15] Patel A., Petrone B., Carter K.R. (2024). Percutaneous Vertebroplasty and Kyphoplasty.

[16] Greenwood T.J., Wallace A., Friedman M.V., Hillen T.J., Robinson C.G., Jennings J.W. (2015). Combined Ablation and Radiation Therapy of Spinal Metastases: A Novel Multimodality Treatment Approach. Pain Physician.

[17] Levy J., Hopkins T., Morris J., Tran N.D., David E., Massari F., Farid H., Vogel A., O’Connell W.G., Sunenshine P. (2020). Radiofrequency Ablation for the Palliative Treatment of Bone Metastases: Outcomes from the Multicenter OsteoCool Tumor Ablation Post-Market Study (OPuS One Study) in 100 Patients. J. Vasc. Interv. Radiol..

[18] Levy J., David E., Hopkins T., Morris J., Tran N.D., Farid H., Massari F., O’Connell W.G., Vogel A., Gangi A. (2023). Radiofrequency Ablation Provides Rapid and Durable Pain Relief for the Palliative Treatment of Lytic Bone Metastases Independent of Radiation Therapy: Final Results from the OsteoCool Tumor Ablation Post-Market Study. Cardiovasc. Interv. Radiol..

[19] Pusceddu C., Marsico S., Derudas D., Ballicu N., Melis L., Zedda S., De Felice C., Calabrese A., Santucci D., Faiella E. (2023). Clinical Rationale of Using Steerable Technologies for Radiofrequency Ablation Followed by Cavity Creation and Cement Augmentation in the Treatment of Painful Spinal Metastases. Curr. Oncol..

[20] Ragheb A., Vanood A., Fahim D.K. (2022). The Addition of Radiofrequency Tumor Ablation to Kyphoplasty May Reduce the Rate of Local Recurrence in Spinal Metastases Secondary to Breast Cancer. World Neurosurg.

[21] Sayed D., Jacobs D., Sowder T., Haines D., Orr W. (2019). Spinal Radiofrequency Ablation Combined with Cement Augmentation for Painful Spinal Vertebral Metastasis: A Single-Center Prospective Study. Pain Physician.

[22] Shawky Abdelgawaad A., Ezzati A., Krajnovic B., Seyed-Emadaldin S., Abdelrahman H. (2021). Radiofrequency ablation and balloon kyphoplasty for palliation of painful spinal metastases. Eur. Spine J..

[23] Georgy B.A. (2009). Bone cement deposition patterns with plasma-mediated radio-frequency ablation and cement augmentation for advanced metastatic spine lesions. Am. J. Neuroradiol..

[24] Gu Y.F., Tian Q.H., Li Y.D., Wu C.-G., Su Y., Song H.-M., He C.-J., Chen D. (2017). Percutaneous vertebroplasty and interventional tumor removal for malignant vertebral compression fractures and/or spinal metastatic tumor with epidural involvement: A prospective pilot study. J. Pain Res..

[25] Jain S., Kinch L., Rana M., Anitescu M. (2020). Comparison of post-operative pain scores and opioid use between kyphoplasty and radiofrequency ablation (RFA) systems combined with cement augmentation. Skelet. Radiol..

[26] Lv N., Geng R., Ling F., Zhou Z., Liu M. (2020). Clinical efficacy and safety of bone cement combined with radiofrequency ablation in the treatment of spinal metastases. BMC Neurol..

[27] Masala S., Roselli M., Massari F., Fiori R., Ursone A., Fossile E., Laudisi A., Simonetti G. (2004). Radiofrequency Heat Ablation and Vertebroplasty in the treatment of neoplastic vertebral body fractures. Anticancer Res..

[28] Munk P.L., Rashid F., Heran M.K., Papirny M., Liu D.M., Malfair D., Badii M., Clarkson P.W. (2009). Combined Cementoplasty and Radiofrequency Ablation in the Treatment of Painful Neoplastic Lesions of Bone. J. Vasc. Interv. Radiol..

[29] Senol N., Oguzoglu A.S., Goksel H.M. (2022). Radiofrequency Ablation and Augmentation in the Management of Spinal Metastases: Clinical Experience in 41 Patients. World Neurosurg..

[30] Reyes M., Georgy M., Brook L., Ortiz O., Brook A., Agarwal V., Muto M., Manfre L., Marcia S., Georgy B.A. (2018). Multicenter clinical and imaging evaluation of targeted radiofrequency ablation (t-RFA) and cement augmentation of neoplastic vertebral lesions. J. Neurointerv. Surg..

[31] Sandri A., Carbognin G., Regis D., Gaspari D., Calciolari C., Girardi V., Masueto G., Bartolozzi P. (2010). Combined radiofrequency and kyphoplasty in painful osteolytic metastases to vertebral bodies. Radiol. Med..

[32] Tomasian A., Hillen T.J., Chang R.O., Jennings J.W. (2018). Simultaneous bipedicular radiofrequency ablation combined with vertebral augmentation for local tumor control of spinal metastases. Am. J. Neuroradiol..

[33] Yildizhan S., Boyaci M.G., Rakip U., Aslan A., Canbek I. (2021). Role of radiofrequency ablation and cement injection for pain control in patients with spinal metastasis. BMC Musculoskelet Disord..

[34] Zheng L., Chen Z., Sun M., Zeng H., Zuo D., Hua Y., Cai Z. (2014). A preliminary study of the safety and efficacy of radiofrequency ablation with percutaneous kyphoplasty for thoracolumbar vertebral metastatic tumor treatment. Med. Sci. Monit..

[35] Wang L., Lu M., Zhang X., Zhao Z., Li X., Liu T., Xu L., Yu S. (2023). Risk factors for pulmonary cement embolism after percutaneous vertebroplasty and radiofrequency ablation for spinal metastases. Front. Oncol..

[36] Wang F., Gu J., Xu C., Li G., Lv P. (2022). The combination of radiofrequency ablation and vertebroplasty shows advantages over single vertebroplasty in treating vertebral neoplastic lesions. Skelet. Radiol..

[37] Proschek D., Kurth A., Proschek P., Vogl T.J., Mack M.G. (2009). Prospective Pilot-study of Combined Bipolar Radiofrequency Ablation and Application of Bone Cement in Bone Metastases. Anticancer Res..

[38] Madaelil T.P., Wallace A.N., Jennings J.W. (2016). Radiofrequency ablation alone or in combination with cementoplasty for local control and pain palliation of sacral metastases: Preliminary results in 11 patients. Skeletal Radiol..

[39] Zhao W., Wang H., Hu J.-H., Peng Z.-H., Chen J.-Z., Huang J.-Q., Jiang Y.-N., Luo G., Yi G.-F., Shen J. (2018). Palliative pain relief and safety of percutaneous radiofrequency ablation combined with cement injection for bone metastasis. Jpn. J. Clin. Oncol..

[40] Kastler A., Barbé D.A., Alemann G., Hadjidekov G., Cornelis F.H., Kastler B. (2021). Bipolar radiofrequency ablation of painful spinal bone metastases performed under local anesthesia: Feasibility regarding patient’s experience and pain outcome. Medicina.

[41] Chen A.L., Sagoo N.S., Vannabouathong C., Reddy Y., Deme S., Patibandla S., Passias P.G., Vira S. (2024). Combination radiofrequency ablation and vertebral cement augmentation for spinal metastatic tumors: A systematic review and meta-analysis of safety and treatment outcomes. N. Am. Spine Soc. J..

[42] Feigl G.C., Dreu M., Kastner M., Rosmarin W., Ulz H., Kniesel B., Likar R. (2017). Thermocoagulation of the Medial Branch of the Dorsal Branch of the Lumbal Spinal Nerve: Flouroscopy Versus CT. Pain Med..

[43] Lee S.A., Chiu C.K., Chan C.Y.W., Yaakup N.A., Wong J.H.D., Kadir K.A.A., Kwan M.K. (2020). The clinical utility of fluoroscopic versus CT guided percutaneous transpedicular core needle biopsy for spinal infections and tumours: A randomized trial. Spine J..

[44] Amoretti N., Marcy P.Y., Lesbats-Jacquot V., Hovorka I., Fonquerne M.-E., Roux C., Hericord O., Maratos Y., Euller-Ziegler L. (2009). Combined CT and fluoroscopic guidance of balloon kyphoplasty versus fluoroscopy-only procedures. Skelet. Radiol..

[45] Wieschhoff G.G., Miskin N.P., Kim J.S., Hamberg L.M., Mandell J.C. (2022). Radiation dose of fluoroscopy-guided versus ultralow-dose CT-fluoroscopy-guided lumbar spine epidural steroid injections. Skelet. Radiol..

[46] Sag A.A., Zuchowski A., Ronald J., Goodwin C.R., Enterline D.S. (2022). Augmented reality overlay fluoroscopic guidance versus CT-fluoroscopic guidance for sacroplasty. Clin. Imaging.

